# Pathogenicity and immune response of turkey A(H1N2) influenza virus of swine-origin on turkeys and mice

**DOI:** 10.1186/s13567-026-01728-8

**Published:** 2026-06-03

**Authors:** Chloé Chavoix, Charlotte Pain-Deloizy, Pascale Massin, Katell Louboutin, Florent Souchaud, Rachel Busson, Carole Guillemoto, Angélina Orosco, Isabelle Pierre, François-Xavier Briand, Fabrice Touzain, Michel Amelot, Alassane Keita, Thibaut Larcher, Jean-Marc Laferte, Eric Niqueux, Audrey Schmitz, Gaëlle Simon, Béatrice Grasland, Ronan Le Goffic

**Affiliations:** 1 Unit Virology, Immunology and Parasitology in Poultry and Rabbits, Anses, Ploufragan-Plouzané-Niort Laboratory, Ploufragan, France; 2 INRAE, UMR892, Unit Molecular Immunology and Virology, Jouy-en-Josas, France; 3 Unit Avian and Rabbit Breeding and Experimental Facility, Anses, Ploufragan-Plouzané-Niort Laboratory, Ploufragan, France; 4 Oniris/INRAE, UMR703, APEX-PAnTher, Nantes, France; 5 ECAM Rennes-Louis de Broglie, Cedex 9, 29128 Rennes, CS France; 6 Unit Swine Virology, Innovation and Genomics, Anses, Ploufragan-Plouzané-Niort Laboratory, Ploufragan, France

**Keywords:** Influenza A virus, H1N2, cross-species transmission, swine, turkey, mouse

## Abstract

In 2020, a new swine influenza H1_av_N2#E genotype emerged in pig farms in France. Shortly after its emergence in swine, this virus was detected in breeding turkey farms, and was responsible of a zoonotic infection. Phylogenetic analyses previously conducted on viral sequences led to the selection of three viruses: the first strain detected in turkeys, resulting from a recent spillover from swine to turkeys (A); a second virus also detected in turkeys and likely adapted to this species (B); and a swine reference strain (C). In the context of species-barrier crossing, the aim of this study was to characterize in vivo these three viruses in an avian model, the breeding turkey, and in a mammalian model, the mouse. Clinical signs, lesions, viral loads, and immune responses were evaluated. In turkeys inoculated experimentally, all three viruses caused similar very mild clinical signs and no viral shedding. In contrast, in mice, virus A caused marked clinical signs associated with mortality, along with a weak inflammatory response. Conversely, virus C caused few clinical signs but showed a more pronounced inflammatory response. Surprisingly, only two nonsynonymous mutations were present between viruses C and A: K189R in PB1 and E233K in HA. Virus B displayed an intermediate profile. There was no difference in genomic load in the lungs among the three viruses, suggesting that the observed differences in pathogenicity were due to the immune response rather than viral replication.

**Supplementary Information:**

The online version contains supplementary material available at 10.1186/s13567-026-01728-8.

## Introduction

Influenza A viruses (IAVs) are known to cause respiratory infections and represent a major concern in both animal and public health. Many species can be infected by IAVs, particularly wild birds considered as natural reservoirs, and domestic poultry. In pigs and humans, active intra-species circulation occurs [1]. IAVs can additionally infect other mammalian species such as horses, domestic and wild carnivores, marine mammals, and cattle [2–4].

The evolutionary mechanisms of IAVs, particularly mutations arising from polymerase errors and the generation of reassortant viruses through genomic segment exchange, can lead to species barrier crossing and adaptation to a new host [5]. Cross-species transmission is a multifactorial process, enabled notably by viral adaptation to host sialic acid (SA) receptors, compatibility of internal proteins, as well as ecological and epidemiological factors related to species proximity [6]. The nature of the linkage between SA and the carbohydrate chains on glycoproteins and glycolipids at the cell surface determines host specificity of the virus [7]. While α-2,3-linked SA are preferentially recognized by avian IAVs, α-2,6-linked SAs are preferred by human IAVs. Pigs possess both types of SA receptors in their respiratory tract. This distribution, which is similar to that of humans, makes pigs a well-recognized “mixing vessel” for avian, human, and swine IAVs [8, 9]. Turkeys also express both α-2,3- and α-2,6-linked SA receptors in their respiratory epithelium and may likewise serve as a host for the generation of reassortant viruses [10]. Cross-species transmission has been regularly observed between different species, for instance between birds and pigs, birds and marine mammals, and pigs and humans [11–13].

However, numerous host restrictions limit the cross-species transmission of IAVs. The host innate immune response plays a key role in restricting transmission, notably through pattern recognition receptors (PRRs) such as retinoic acid-inducible gene I (RIG-I) [14]. Recognition of viral RNA by RIG-I activates the interferon response, leading to the production of antiviral proteins such as myxovirus (Mx) resistance, which targets the IAV nucleoprotein (NP) by blocking its nuclear import [15, 16]. The efficacy of this mechanism varies among species [17]. The IAV polymerase complex proteins (PB2, PB1, and PA) require host-specific conditions to support viral replication, including body temperature, which differs across species [18]. In addition, host-specific cellular cofactors, such as proteins of the acidic nuclear phosphoprotein 32 (ANP32) family, interact with the viral polymerase complex, and their compatibility is essential for efficient replication [19]. These various host restriction factors, and host-specific cofactors, which act at different levels and stages of infection, vary between species and constitute critical barriers to IAV cross-species transmission.

Since 2020, several cross-species transmission events of swine-origin IAVs to turkeys have been reported in France. A new IAV genotype named H1_av_N2#E emerged in pig farms in Brittany where it became the predominant circulating genotype [20]. In parallel, the same virus was detected in around 20 breeding turkey flocks in Brittany, all of which showed marked drops in egg production. In 2021, another cross-species transmission event involving this virus was reported, this time from pigs to a human [21]. Phylogenetic analyses of the viral sequences detected in turkeys and pigs suggested that at least nine independent and successive cross-species transmission events from pigs to turkeys occurred [22]. These analyses also indicated that the virus may have circulated among turkey flocks, highlighting the potential for viral selection and adaptation in this host. These phylogenetic analyses allowed the selection of three strains, selected on the basis of their host species of origin and degree of host adaptation. The first one, named Swine virus, was detected in June 2020 in a swine farm in the department of Ille-et-Vilaine. This parental swine H1_av_N2#E virus served as a reference, as it had already been studied after experimental infection in swine, a species in which the disease was reproduced as expected [23]. The two other viruses were detected in turkeys. The second one, named Turkey swine-like virus, was detected slightly earlier, in April 2020, in a turkey farm in the department of Côtes-d’Armor. This was the first reported case detected in turkeys, resulting from a recent cross-species transmission from swine to turkeys. This virus was therefore phylogenetically very close to the Swine virus. Finally, the third strain named Turkey virus was detected about one and a half years later, in October 2021, in a turkey farm in the Côtes-d’Armor district. This virus appeared to belong to a cluster composed of several sequences only identified in turkeys, suggesting ongoing circulation within this species [22]. In vitro characterization of these three viral isolates on different cell types revealed similar replication patterns, with minor differences. These findings show off the importance of further characterization of these cross-species transmissible viruses using more complex models.

The objective of this study was to characterize in vivo the pathogenicity and the induced immune response of H1_av_N2#E viruses using two animal models: an avian model (breeding turkey) and a mammalian model (mouse). The breeding turkey was considered as a relevant model as it directly reflects the field situation. It should allow a faithful characterization of the viruses detected in turkey farms. Although not a natural host for IAV, the mouse was chosen as a suitable surrogate mammalian model for studying these viruses. It is a well-established model known to be susceptible to IAV infections [24, 25] and an easy-to-use model. This study focused on characterizing the pathogenicity of three H1_av_N2#E viruses in these two species. Clinical signs, viral shedding, induced immune responses, and histopathological lesions were assessed and compared between these three selected viral strains in these two different species.

## Materials and methods

### Selection and multiplication of the viral strains

The Swine virus (A/swine/France/35–200154/2020, accession numbers: MZ088854 to MZ088861) was provided by the French National Reference Lab for Swine Influenza (Anses, Ploufragan, France) and multiplied in Madin-Darby Canine Kidney (MDCK) cells as previously described [23]. It was then multiplied through two successive passages on MDCK cells. The Turkey swine-like virus (A/turkey/France/20P005076/2020, accession numbers: EPI4543932 to EPI4543939) and the Turkey virus (A/turkey/France/21P011515/2021, accession numbers: EPI4544020 to EPI4544027) were isolated and propagated in 12-day-old specific-pathogen-free (SPF) embryonated chicken eggs, then further propagated in MDCK cells with two successive passages as previously described [22].

### Quantification of infectious viral loads for both trials

All three viruses were titrated in tissue culture infectious dose for 50% of the cells (TCID_50_) per mL, as previously described [22]. Quantification of infectious titers in plaque forming units (PFU) per mL was also performed. Cell culture plates (six-wells) were seeded 24 h before titration with 1 × 10^6^ MDCK cells per well in a volume of 2 mL of complete cultured medium, consisting of Minimum Essential Medium (MEM; Gibco, ThermoFisher Scientific, Waltham, MA, USA), supplemented with 5% of decomplemented fetal calf serum (FCS) (Eurobio Scientific, Les Ulis, France), antibiotics (100 U/mL of penicillin and 1 mg/mL of streptomycin, Sigma-Aldrich Merck, Darmstadt, Germany), and 0.01 M of tricine (Sigma-Aldrich Merck, Darmstadt, Germany). The samples to be titrated were serial tenfold diluted (10^–2^ to 10^–7^) in Dulbecco’s modified Eagle’s medium (DMEM; Gibco, ThermoFisher Scientific) supplemented with antibiotics. Cells were washed twice with the same medium and then inoculated in duplicate for each dilution for 1 h at 37 °C with 5% CO_2_, with gentle agitation every 10 min. The inoculum was removed and the cells were incubated for 72 h at 37 °C with 5% CO_2_ in DMEM supplemented with 1% L-glutamine (0.2 M; Gibco, Invitrogen, Thermo Fisher Scientific), 0.375% sodium bicarbonate (Gibco, Invitrogen, Thermo Fisher Scientific), 1% agarose (Invitrogen, Thermo Fisher Scientific), 1.8 µg/mL N-tosyl-L-phenylalanine chloromethyl ketone-treated trypsin (TPCK-treated trypsin; Worthington Biochemical Corporation, Lakewood, NJ, USA), and antibiotics. After 72 h, the DMEM/agarose overlays were removed, the cells were fixed with a crystal violet solution and then rinsed thoroughly with bleach followed by running water. Plaques were counted, and PFU titers were determined by averaging the two replicates.

The infectious titers obtained for the viral stocks were 1.31 × 10^6^ TCID_50_/mL (3.25 × 10^6^ PFU/mL) for the Turkey swine-like virus, 3.89 × 10^6^ TCID_50_/mL (1.76 × 10^7^ PFU/mL) for the Turkey virus, and 6.31 × 10^5^ TCID_50_/mL (2.39 × 10^5^ PFU/mL) for the Swine virus. For the higher inoculum dose administered to mice for the Swine virus, the infectious titer was insufficient. A second vial, provided by the French National Reference Laboratory for Swine Influenza and exhibiting a higher titer, was used. The new infectious titer was 1.95 × 10^7^ TCID_50_/mL and the plaque assay was not performed. The viral titer in PFU/mL was estimated from the TCID_50_/mL value using the Poisson distribution approximation, in which the mean infectious dose at 50% infection corresponds to ln(2) = 0.693 infectious units per well [26]. The formula PFU ≈ TCID_50_ × 0.70 yielded an estimated PFU titer of 1.37 × 10^7^ PFU/mL.

The virus from viral stocks were sequenced by next-generation sequencing (NGS), as previously described [22]. Concerning amino acid numbering, the first amino acid was the first coding methionine. The HA (Hemagglutinin) numbering was on the basis of the first viral sequence of influenza H1_av_N2#E detected in swine in February 2020 in Brittany, A/swine/France/56–200050/2020 (accession numbers: MZ088174 to MZ088181) [20].

### Ethical statement

This study was carried out in accordance with Anses and INRAE guidelines in compliance with European animal welfare regulation. Mice and turkey protocols were approved by the respective ethic committees of INRAE and Anses, COMETHEA and COMETH, under relevant institutional authorization (Ministère de l’éducation nationale, de l’enseignement supérieur et de la recherche) with authorization numbers: APAFIS no. 41483–2023030717306691 v8, APAFIS no. 22729–2019040213398185, and APAFIS no. 49618–202405209027279.

### Turkey trial and sample collection

All turkey experiments were conducted in negatively pressurized biosafety level 2 (BSL2) animal facilities at the Anses Laboratory of Ploufragan-Plouzané-Niort. The turkeys used in this study were 34-week-old SPF breeding females. They originated from the SPF turkey flock of Anses. All turkeys were acclimated for 1 month before the beginning of the experimental procedure. Throughout the experiment, water and feed were provided *ad libitum*. To monitor egg laying, plastic nests filled with wood shavings were implemented. To ensure optimal laying conditions, a lighting program of 140 lux for 14 hours per day was implemented. The 24 turkeys were divided into four groups. Three groups of seven birds each were inoculated respectively with the Turkey swine-like virus, the Turkey virus, or the Swine virus. A control group of three turkeys received a mock-inoculation with phosphate-buffered saline (PBS). Each bird was inoculated via ocular and nasal routes with 160 µL PBS containing 10^5^ TCID_50_ of virus or with PBS alone. Animals were weighed at the beginning and at the end of the procedure. The trial lasted for 10 days. Individual oropharyngeal and cloacal swabs were collected four days before infection, and at 1, 2, 3, 4, 7 and 10 days post-infection (dpi). Blood samples were collected from the occipital sinus at −4, 4, 7, and 10 dpi. At 4 and 10 dpi, euthanasia was performed by electronarcosis followed by exsanguination. Tissue samples were collected from the trachea, lungs, liver, intestine, cecum, kidney, and oviduct for virological analyses. For each animal, daily clinical observations were performed along with egg-laying monitoring. Observations included general (apathy, somnolence, prostration), respiratory (discharge, conjunctivitis, cough/rales, head/sinus swelling), neurological (abnormal gait, torticollis), and cutaneous signs (cyanosis, petechiae), as well as any other abnormalities. To provide a synthetic representation of clinical intensity within groups, a score was assigned to clinical signs for which a consistent weighting could be defined retrospectively on the basis of their estimated functional impact. The signs included were partially closed eyes (score = 1); rales on a 3-level scale: mild (score = 1), moderate (score = 2), and marked (score = 3); labored breathing (score = 4); and apathy (score = 2). For each group and time point, individual scores were summed. The theoretical maximum score per animal was 10 and the average clinical score per animal was plotted.

### Turkey serology

The detection of anti-NP antibodies in the sera was performed using an indirect NP-ELISA (ID Screen^®^ Influenza A Nucleoprotein Indirect kit, Innovative Diagnostics, Grabels, France) according to the manufacturer’s instructions. After color development, the optical density (OD) of the wells was measured at a wavelength of 450 nm using a microplate reader (Tecan, Männedorf, Switzerland) to quantify antibody levels. The sample-to-positive corrected ratio (S/P ratio) was calculated using the OD values of the positive and negative control sera provided in the kit, according to the following formula:

$${S/P ratio}_{sample}=\frac{{OD}_{sample}- {OD }_{mean of negative controls} }{O{D }_{mean of positive controls}- {OD }_{mean of negative controls}}$$

This ratio was plotted graphically. An S/P ratio ≥ 0.5 was considered indicative of a positive sample.

### Mouse trial and sample collection

All animal experiments were conducted in negatively pressurized BSL2 animal facilities at INRAE in pathogen-free conditions, within the Infectiology of Fishes and Rodents Facilities unit (IERP-UE907, Jouy-en-Josas Research Center, France). BALB/c female mice were purchased from the Centre d’élevage R. Janvier (Le Genest Saint-Isle, France) and NF-κB–luciferase transgenic BALB/C mice were bred locally in IERP-UE907 rodent facilities. All mice were used at about 8 weeks of age. All mice were acclimated for 1 week before the beginning of the procedure. Mice were fed with normal mouse chow and water *ad libitum* and were reared and housed under standard conditions with air filtration.

Two inoculum doses, one low and one high, were administered. The highest infectious dose corresponded to the LD_50_ (lethal dose for 50% of the mice) of the Turkey virus. The lowest infection dose was chosen with a difference of at least one log_10_ compared with the highest dose, while still ensuring the observation of clinical signs. For each dose, four independent groups of 12 animals were established: one group inoculated with the Turkey swine-like virus, one with the Turkey virus, one with the Swine virus, and one control group that received a mock-inoculation with PBS. For inoculation, mice were lightly anesthetized by intraperitoneal injection of 150 µL containing a mixture of ketamine and xylazine (60 and 12 mg/kg, respectively) to minimize suffering. They were inoculated intranasally with 1.2 × 10^4^ or 1.4 × 10^5^ PFU of virus in 50 µL of PBS for the lowest or the highest infectious doses, respectively. For the NF-kB–luciferase transgenic mice, only the highest infectious dose was used. Control mice received the same treatment as infected mice but were mock-inoculated with PBS only.

The experiment lasted for 15 days. Daily monitoring included body weight, rectal temperature (BIO-TK9882; Bioseb Instruments), clinical score, and mortality. For clinical scoring, several parameters were assessed and graded from 0 (none) to 3 (severe): weight loss, fecal appearance, presence of anal bleeding, body temperature, coat condition, posture, response to stimuli, activity/locomotion, dehydration, and eye and ear appearance. The clinical score for each animal was the sum of the scores assigned to these parameters. For each condition and day, the mean clinical score was calculated from all individuals and a cumulative clinical score was calculated by summing the daily mean scores across days. This cumulative clinical score was plotted graphically. Humane endpoints were used during infectious study: mice were euthanized by cervical dislocation when body weights were reduced to 80% of the starting weights or in case of a non-transient decrease in whole-body temperature below 32 °C. In addition, animals that reached moribund state (unresponsive and unaware of stimuli) were also euthanized. For the highest dose, buccal swabs were performed at 1, 3, 6, 8, 10, and 14 days and processed in the same way as previously described for oropharyngeal and cloacal swabs in turkeys in order to determine the viral genomic loads.

For the lowest infectious dose experiment, lungs were collected at 4, 7, and 15 days post infection (dpi) from four animals of each group following cervical dislocation. Bronchoalveolar lavages (BALs) were also performed at these time points by injecting 1 mL of PBS into the lungs via the trachea and collecting the fluid. One milliliter of blood was collected by the vena cava. After centrifugation at 300 *g*, the resulting serum was stored. For the highest infectious dose experiment, lungs, olfactory bulbs, blood, and BALs were collected at 3, 7, and 15 dpi from four animals of each group following cervical dislocation. For both doses, the left lung was fixed in 4% paraformaldehyde (PFA) for histological analyses. The right lung and the olfactory bulbs (highest dose only) were frozen at −80 °C for RNA extraction.

### In vivo imaging system (IVIS) monitoring

Bioluminescence measurements of the NF-κB–luciferase transgenic mice were measured using the IVIS Spectrum imaging system (PerkinElmer) following previously established protocols (Mettier et al. 2021) at 3, 7, and 10 dpi. Mice were anesthetized, and luminescence was measured 5 min after intravenous injection of 50 μL of PBS containing D-luciferin (0.75 mg kg^−1^; Sigma). Living Image software (version 4.0; PerkinElmer) was used to measure luciferase activities. Bioluminescence images were acquired for 1 min with f/stop = 1 and binning = 8. A digital false-color photon emission image of the mouse was generated, and photons were counted within the chest area. Photon emission was measured as radiance in p s^−1^ cm^−2^ sr^−1^.

### Whole-body plethysmography

Respiratory measurements were acquired by using a whole-body plethysmograph (EMKA) as previously described [27]. For the lowest infectious dose, measures were taken at 2, 3, 7, and 14 dpi. For the highest infectious dose, measures were performed at 2, 3, 6, 8, 10, and 14 dpi. Briefly, conscious mice were placed in chambers where breathing-induced changes in differential pressure were continuously measured. After a few minutes of acclimatization, ten measurements were recorded, each consisting of an average over ten respiratory cycles. Lung function parameters were analyzed with a particular focus on enhanced pause (Penh), a derived indicator of respiratory distress calculated over a full breathing cycle. Penh is defined by the following formula:

$$Penh=\frac{PEF}{PIF} \mathrm{x} \frac{(Te-Tr)}{Tr},$$

where PEF is the peak expiratory flow of breath, PIF is the peak inspiratory flow of breath, Te is the expiratory time, and Tr (relaxation time) is the time required to exhale 65% of the breath volume [28]. Penh is a robust indicator of respiratory distress during IAV infections, especially in the early phase of infection [29].

### Cytospin of BALs and cell counts

BALs were centrifuged at 2000 rpm for 10 min at 4 °C within a maximum of 3 h after collection. Supernatants were transferred for infectious titer determination and myeloperoxidase (MPO) activity assay. Cell pellets were resuspended in 200 µL of PBS and counted using a Kova slide. Approximately 100,000 cells were centrifuged onto a slide, then fixed for 20 min with 2% paraformaldehyde (PFA) and stained with May–Grünwald Giemsa (MGG). Cell counting and classification were performed using an artificial-intelligence-based model built on neural network learning. Three immune cell types were included in the analysis, grouped into two cell categories: mononuclear cells with macrophages and lymphocytes, and polymorphonuclear cells with neutrophils. Digitized slide images in MRXS format (25,600 × 25,600 pixels) were subdivided into smaller images and converted to PNG format (640 × 640 pixels). A total of 250 images including 589 macrophages, 889 lymphocytes, and 546 neutrophils were labeled using the LabelImg software. Image rotation for data augmentation increased the number of labeled cells fourfold. For each cell, labeling required drawing a rectangular bounding box around the cell and indicating the corresponding cell type. An application named CytoClass^®^ was developed to perform cell type recognition and counting across the entire cell spot on the cytospin slides. It is a user interface that enabled the analysis of a whole-slide image within minutes, based on YOLO and following model training. For each individual sample, the total number of each cell category and their respective proportions were estimated using the application. The relative proportions of mononuclear and polymorphonuclear cells for the three viruses at 3, 7, and 15 dpi were graphically represented.

### Histopathological analyses and immunostaining of mouse lungs

Left lungs were fixed for 24 h in 4% PFA immediately following euthanasia. They were then rinsed into 70% ethanol and stored at 4 °C. Tissues were dehydrated through successive baths of ethanol ranging from 70% to 100%. Tissues were embedded in paraffin, sectioned at 5 µm thickness using a microtome (Leica), and mounted onto slides. Sections were stained with hematoxylin–eosin–saffron (HES) for histological analysis. The slides were digitized in MRXS format and examined using the open-source image analysis software QuPath (v.0.5.1) [30]. Histological assessments were performed by a trained European board-certified in pathologic anatomy veterinary pathologist. Lung lesions were scored on the basis of eight criteria with 3- or 4-level scales (Table 1).

**Table 1:** **Criteria and scores of histopathological lung lesions**

	Scores			
Histological criteria	0	1	2	3
Bronchial epithelium damage	No lesion	Small clusters of necrotic epithelial cells	Scattered foci of epithelial degeneration with layer architecture partial loss	Focal complete epithelial loss
Bronchial content	Empty lumen	Presence of a small amount of material	Partial obliteration	Complete occlusion
Vascular wall changes	No lesion	Leukostasis	Focal wall damages including leukocytoclasis	Transmural vessel wall alteration and/or vascular lumen obliteration
Perivascular edema	No lesion	Focal mild edema	Extended marked edema with lymphoid vessel dilatation	–
Peribronchial/perivascular cuffing	No lesion	Focal inflammatory cell infiltration	Circumferential inflammatory cell infiltration	Coalescing inflammatory cell infiltration between bronchi and vessels
Interstitial pneumonia	No lesion	Focal and mild alveolar wall thickening	Extended and/or severe alveolar wall thickening	–
Alveolar content	No lesion	Mild increase in number of intra-alveolar cells	Intra-alveolar clumps of inflammatory cells	Alveolar lumen filling by inflammatory cells
Emphysema	No lesion	Dilated and coalescent alveoli	“Bullae” in the parenchyma	–

A cumulative score was then calculated by summing the individual scores with a total possible range from 0 to 22. This cumulative score was plotted graphically. A score above 12 was classified as severe.

To visualize viral localization in the lungs, anti-NP immunostaining was performed. Lung sections were incubated overnight at 4 °C with a polyclonal primary antibody targeting the IAV nucleoprotein NP (Rabbit anti NP polyclonal, 1:200 dilution, Invitrogen, Carlsbad, USA). After three washes, sections were incubated with a secondary antibody conjugated to AF488 (Goat anti rabbit IgG AF488, 1:800 dilution, SouthernBiotech, Birmingham, USA). Cell nuclei were counterstained with Hoechst dye (Hoechst 33,342, 1:1000 dilution, Invitrogen). Imaging was conducted using the Pannoramic Scan system (3DHISTECH).

Image analysis from whole lung sections was performed using ImageJ software (version 1.54d). For each image, the scale was manually defined on the basis of the integrated scale bar. Red/green/blue (RGB) channels were separated using the split channels function to independently analyze the blue (Hoechst) and green (NP) signals. For the blue channel, a fixed intensity threshold (range: 40–255) was manually applied to detect nuclei. This threshold was determined visually by testing a representative set of images (*n* = 10) and was chosen as the one that best matched the Hoechst-stained areas visible in the original composite image. The Otsu automatic thresholding method was applied to further refine the binary segmentation, as it allows optimal separation between background and signal pixel populations. The NP signal was processed similarly. A fixed intensity threshold (range: 75–255) was determined visually by testing a representative set of images (*n* = 10) to isolate specific NP signal while excluding background noise. The Otsu method was also applied. Following thresholding, signal quantification from images was processed using the analyze particles function. Detection parameters were adjusted to display identified objects as overlays and to quantify the total area occupied by each signal of interest. Viral load was expressed as the ratio of NP-positive area to Hoechst-positive area, corresponding to the percentage of infected tissue normalized to nuclear density. This normalized ratio was plotted for each experimental condition.

### Transcriptomic analyses in mice lungs

Transcriptomic analyses were performed on RNA extracted from the lungs of control mice and of mice inoculated with the highest infectious dose. The expression of 11 inflammation-related genes was quantified. The gene names and the corresponding primer sequences are summarized in Table 2.

**Table 2:** **Details of the genes and primers used for transcriptomic analyses**

Gene	Forward primer	Reverse primer
β-actin	CCTTCTTGGGTATGGAATCCTGT	CACTGTGTTGGCATAGAGGTCTTTAC
β-tubulin	CAACTTTATCTTTGGTCAGAGTGG	CAGGCAGTCACAATTCTCAC
HPRT	TGACACTGGCAAAACAATGCA	GGTCCTTTTCACCAGCAAGCT
CCL5	ATCTTGCAGTCGTGTTTGTCA	TCCTAGCTCATCTCCAAATAGTTG
CXCL1	AGACCATGGCTGGGATTCAC	TCGCGACCATTCTTGAGTGT
CXCL10	GCCGTCATTTTCTGCCTCAT	GCTTCCCTATGGCCCTCATT
IFN-α	CTAGGCTCTGTGCTTTCCTG	CTCTTGTTCCTGAGGTTATGAGTC
IFN-λ	ATGCCATCGTGAGTCTCCCTC	GGCTGAGGTTAGCCCACTCTA
IL1-β	TTTGACAGTGATGAGAATGACC	AATGAGTGATACTGCCTGCC
IL6	CTGGGAAATCGTGGAAATGAG	TTTCTGCAAGTGCATCATCG
MxA	GTGGTAGTCCCCAGCAATGT	TGCTGACCTCTGCACTTGAC
NF-κB	GAAATTCCTGATCCAGACAAAAAC	ATCACTTCAATGGCCTCTGTGTAG
RELB	CTTTGCCTATGATCCTTCTGC	GAGTCCAGTGATAGGGGCTCT
TNF-α	ATCGGTCCCAAAGGGATGA	GGTGGTTTGCTACACGTG

Following RNA extraction, samples were treated with DNase using the RQ1 RNase-free DNase provided in the RiboMAX transcription kit (Promega Corporation, Fitchburg, USA). Complementary DNA (cDNA) synthesis was performed using the iScript Advanced cDNA Synthesis Kit for RT-qPCR (Bio-Rad) from 500 ng of RNA dosed by fluorescence with Qubit™ (Invotrogen, ThermoFisher Scientific). The reverse transcription program was set as follows: 46 °C for 20 min followed by 95 °C for 1 min. The resulting cDNA was diluted 1:10 prior to use. Quantification of inflammation-specific messenger RNA (mRNA) expression was carried out by SYBR Green-based qPCR using the Power SYBR™ Green PCR Master Mix kit (ThermoFisher). PCR cycling conditions included an initial denaturation step at 95 °C for 10 min, followed by 40 cycles of 15 s at 95 °C (denaturation) and 1 min at 60 °C (annealing/extension). A melt curve analysis was performed from 60 °C to 95 °C, increasing by 0.5 °C every 5 s to confirm the specificity of primer amplification and the presence of a single PCR product. All qPCR reactions were run on a CFX Connect thermocycler (Bio-Rad), and data were analyzed using Bio-Rad CFX Manager software (version 3.1). Expression was quantified using the ΔΔCt method, normalizing each sample to the geometric mean of three housekeeping genes (HKGs): β-actin, β-tubulin, and HPRT. Relative mRNA levels were calculated versus control mice as 2^−ΔΔCt^ (fold-change) and results were reported as log_2_(fold-change) (i.e., −ΔΔCt).

Heatmaps were generated using the pheatmap function in R software. Hierarchical clustering was applied to the genes (columns) on the basis of Euclidean distance to compare gene expression profiles across conditions (inoculated viruses and sampling times). For two genes *x* and *y*, each represented by their expression values across *n* conditions, the distance *d(x,y)* was defined as:

$$d\left(x,y\right)=\sqrt{{\sum_{i=1}^{n}({x}_{i}-{y}_{i})}^{2}},$$

where *x*_*i*​_ and *y*_*i*_ were the expression values of genes *x* and *y* under condition *i*. This distance reflected the overall difference in expression between two genes across all conditions. Cluster formation was performed using the complete linkage method, which defined the distance between two clusters as the largest distance between any two individual genes, one from each cluster.

### Viral genomic load quantification for both trials

Total RNA from organs and swab samples collected from turkeys was extracted using the KingFisher automated system and the ID Gene™ Mag Fast Extraction Kit (Innovative Diagnostics, Grabels, France). Total RNA from mouse organs were extracted using TRI Reagent according to the manufacturer’s instructions (Molecular Research Center, Cincinnati, USA). Quantification of viral genomes was performed using a TaqMan real-time reverse transcriptase quantitative polymerase chain reaction (rRT-qPCR) assay developed to amplify specifically the HA-1C.2.4 gene of swine IAV strains belonging to the H1_av_N2#E lineage as previously described [20]. The QuantiTect Virus Kit (Qiagen, Hidlen, Germany) was used. All rRT-qPCRs were performed on a CFX Connect thermocycler (Bio-Rad, Hercules, USA) and data analysis was carried out using the Bio-Rad CFX Manager software (version 3.1). An HA gene RNA transcript was generated after TOPO TA cloning by in vitro transcription (ThermoFisher Scientific, Waltham, USA) and used in each run of rRT-qPCR as a standard for viral genome quantification.

### Infectious titer assays and myeloperoxidase (MPO) quantification

The clarified homogenates of mouse lungs inoculated with the highest infectious dose were titrated in TCID_50_ per mL of viral suspension as described previously [22]. The PFU titration of BAL fluids collected from mice was performed as described above but in 12-well culture plates seeded with 4 × 10^5^ MDCK cells per well. Plaque sizes were also measured (*n* = 53 for the Turkey swine-like virus plaques, *n* = 33 for the Turkey virus plaques, and *n* = 28 for the Swine virus plaques). MPO activity was quantified in BAL fluids by incubating samples for 15 min at room temperature with 1 mg/mL o-dianisidine diluted in 0.04% H_2_O_2_. The reaction was stopped by adding a 1% NaN_3_ solution. Absorbance was measured at 460 nm, with an average of nine readings per well and plotted on a graph.

### Statistical analyses

Figures were generated using GraphPad Prism (version 8.0.1) and R Studio (version 2024.12.0) software. For infectious titers and genomic loads in lungs, infectious titers in BAL fluids, and plaque sizes in mice, box-and-whisker plots were constructed using five values: minimum, first quartile, median, third quartile, and maximum. Statistical analyses were performed using one-way analysis of variance (ANOVA) followed by pairwise comparisons using Tukey’s method. Mean values for weight loss, respiratory distress, and NF-κB activity were represented with error bars corresponding to the standard error of the mean (SEM). Cumulative clinical scores in mice were obtained by summing individual scores for all animals within the same condition. For serology in turkeys, histopathological cumulative composite scores, normalized NP area from immunostaining and MPO activity in BALs in mice, individual values were plotted with means associated with error bars corresponding to standard deviation. The proportions of BAL cell types in mice were represented using stacked histograms with error bars corresponding to standard deviations.

Statistical analyses involving two groups were performed using Mann–Whitney tests. Analyses involving three or more groups were performed using Kruskal–Wallis tests followed by Dunn’s multiple pairwise comparisons tests. Differences between groups were considered statistically significant (and the null hypotheses rejected) if the corresponding tests were associated with *p*-values ≤ 0.05. One asterisk indicated a *p*-value ≤ 0.05, two asterisks indicated a *p*-value ≤ 0.01, and three asterisks indicated a *p*-value < 0.001.

## Results

### Overview and characteristics of the three viral strains

The three viral strains had previously been studied in vitro in cell culture and *in ovo* in embryonated chicken eggs [22]. The Turkey swine-like virus and the Swine virus were very close, with only two nonsynonymous mutations in the whole genome: K189R in the PB1 protein and E233K in the HA protein. The Turkey virus showed somewhat greater divergence compared with the first two viruses, with 34 and 35 nonsynonymous mutations compared with the Turkey swine-like virus and the Swine virus, respectively. NGS analyses of the three viral stocks produced to constitute the inocula showed that two mutations emerged in the Turkey virus in the HA protein after a single multiplication on MDCK cells: K233M and E236G. Initially, at these two positions, the Turkey virus harbored the same amino acids as the Turkey swine-like virus (Additional file 1).

### Clinical signs, viral genomic loads, and seroconversion in turkeys

**Figure 1:**
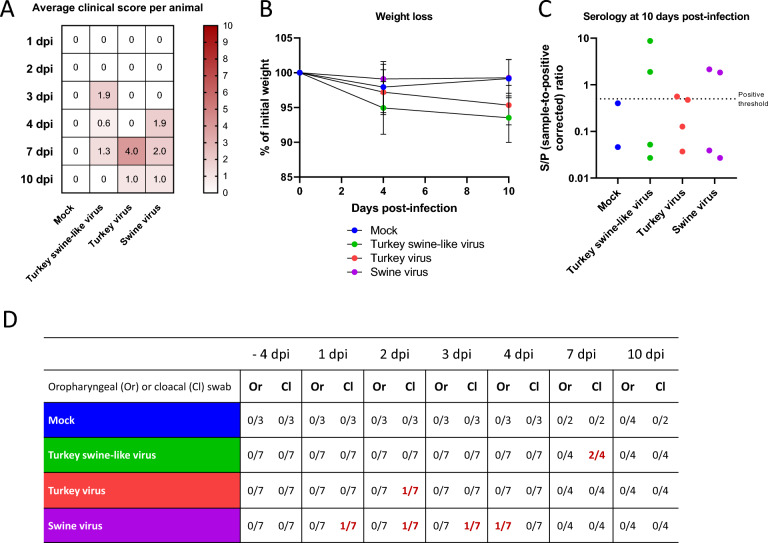
**Clinical signs, seroconversion, and viral genomic load in infected breeding turkeys.** Turkeys were infected with 10^5^TCID_50_ of various viral isolates per animal: Turkey swine-like virus (green), Turkey virus (red), Swine virus (purple), or mock-infected (blue). From −4 dpi to 4 dpi: *n* = 7 per infected group and *n* = 3 for the mock condition, and at 7 and 10 dpi: *n* = 4 per infected group, *n* = 2 for the mock condition. **A** Average clinical scores (partially closed eyes, rales, labored breathing, and apathy) per animal within a group following days post infection, total up to 10. **B** Percentage of initial weight of the turkeys following days post infection. **C** Detection of anti-NP antibodies using S/P ratio. All samples with S/P ≥ 0.5 (dashed line) were considered to be positive samples. Data are presented as mean ± SD. The three points circled in black correspond to the individuals with positive swabs. **D** rRT-qPCR positive oropharyngeal (Or) or cloacal (Cl) swab in turkeys following the days post-infection.

Turkeys from the three inoculated groups showed some respiratory clinical signs (rales and labored breathing), as well as some signs of apathy (Figure 1A). In the group infected with the Turkey swine-like virus, the first clinical signs appeared at 3 dpi with an average clinical score of 1.9/10. Four out of seven turkeys showed a rales score of 2 on a 3-level scale, one out of seven turkeys showed labored breathing, and one out of seven turkeys had abnormal closed eyes. These clinical signs decreased at 4 dpi with an average clinical score of 0.6/10. At 7 dpi the score was 1.3/10, and at 10 dpi, no turkey showed clinical signs. In the group infected with the Turkey virus, clinical signs appeared later, with an average clinical score of 4/10 at 7 dpi. All (4/4) turkeys showed labored breathing. At 10 dpi the score was 1/10 with one out of four turkeys still showing labored breathing. Finally, in the group infected with the Swine virus, clinical signs appeared at an intermediate time point (4 dpi) with an average clinical score of 1.9/10. Two out of seven turkeys showed rales scores of 1 and 2 on a 3-level scale, two out of seven turkeys showed labored breathing, and one out of seven turkeys showed apathy. At 7 dpi, the average clinical score was 2/10 with two out of four turkeys showing a rales score of 1 and 3 on a 3-level scale, and two out of four turkeys showing apathy. At 10 dpi, the score was 1/10 with one out of four turkeys showing respiratory difficulties. In this group, turkeys did not lose weight. No drop in egg production was observed in any of the three groups.

Turkeys infected with the Turkey swine-like virus lost weight, but not significantly compared with controls and to turkeys infected with the Swine virus (Figure 1B). They lost an average of 5.1% of their initial weight at 4 dpi and 6.5% at 10 dpi. In the group infected with the Turkey virus, the animals exhibited slightly less weight loss, still not significantly compared with controls and to turkeys infected with the Swine virus. They lost an average of 2.8% of their initial weight at 4 dpi and 4.7% at 10 dpi.

No NP seroconversion was demonstrated before 10 dpi. Regarding anti-NP antibody production at 10 dpi (Figure 1C) in the group infected with the Turkey swine-like virus, two out of four turkeys tested positive in NP-based indirect ELISA. In the group infected with the Turkey virus, one out of four turkeys tested positive, and in the group infected with the Swine virus, two out of four turkeys tested positive.

Viral genomic detection in organs at 10 dpi, showed no positive results in any of the three groups (data not shown). In oropharyngeal and cloacal swabs, very few detections were observed in all three groups, with late Ct values: 41.9 to 37.1, corresponding to values ranging from 6.1 × 10^2^ to 1.7 × 10^4^ HA gene copies/mL of swab supernatant, respectively (Figure 1D). In the group infected with the Turkey swine-like virus, only two out of four cloacal swabs were positive at 7 dpi. In the group infected with the Turkey virus, only one out of four cloacal swabs was positive at 1 dpi. Finally, in the group infected with the Swine virus, one out of seven cloacal swabs was positive at 1, 2, and 3 dpi, and one out of seven tracheal swabs was positive at 4 dpi.

### Comparison of virulence in mice using low and high infectious dose

**Figure 2:**
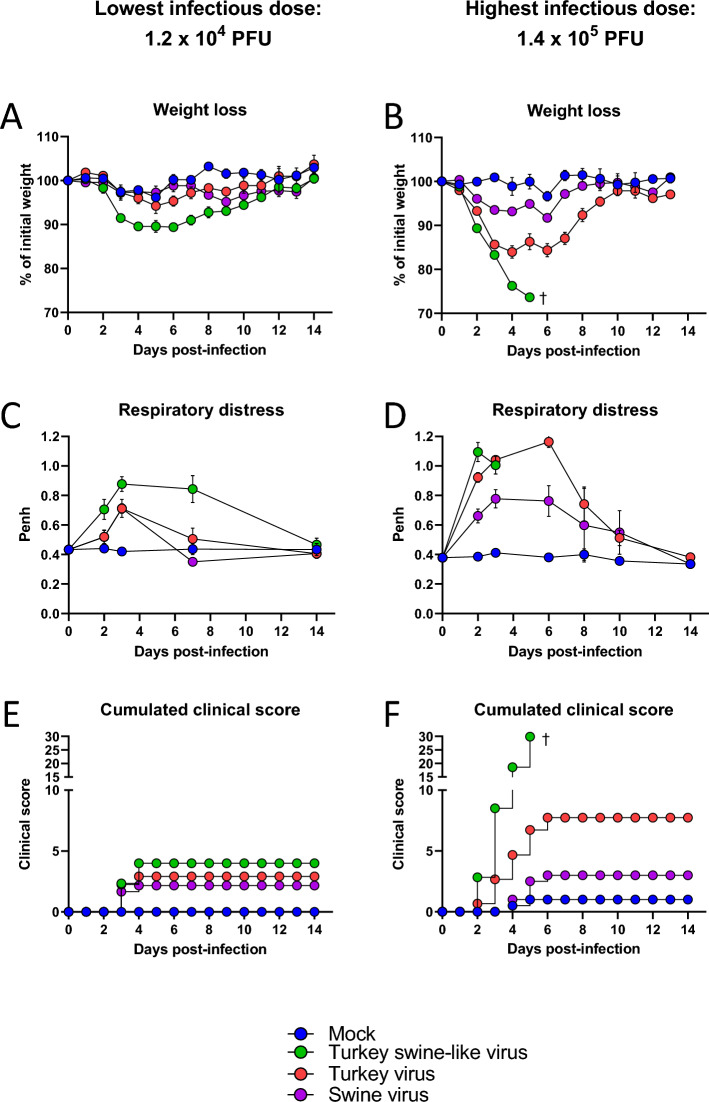
**Weight loss, respiratory distress, and cumulated clinical scores in infected BALB/c mice.** Mice (*n* = 12 per group) were intranasally infected with either the lowest (1.2 × 10^4^ PFU, left panels) or the highest (1.4 × 10^5^ PFU, right panels) infectious dose of various viral isolates: Turkey swine-like virus (green), Turkey virus (red), Swine virus (purple), or mock-infected (blue). For weight loss (**A**, **B**) and clinical scores (**E**, **F**): from 0 to 3 dpi, *n* = 12; from 4 to 7 dpi, *n* = 8 22; and from 8 to 14 dpi, *n* = 4. For respiratory distress (**C**, **D**): from 0 to 6 dpi, *n* = 6 and from 7 to 14 dpi, *n* = 4. The cumulated clinical scores (**E**, **F**) are the cumulated sums of the daily mean clinical scores. ^†^Indicates death of mice in the Turkey swine-like virus condition. Data are presented as mean ± SD.

To compare the virulence of the three different viral strains in a mammalian model, we infected BALB/c mice with either the lowest or the highest infectious dose of each virus. As expected, morbidity was correlated with the administered dose, as shown by the weight loss curves following low-dose (Figure 2A) and high-dose (Figure 2B) infections. At the lowest dose, the Turkey swine-like virus induced a noticeable but transient weight loss (10%) peaking at 5 dpi (Figure 2A), accompanied by increased respiratory distress (Figure 2C) and moderate clinical scores (Figure 2E). The Turkey virus caused a milder phenotype, with moderate weight loss, limited respiratory symptoms, and a lower clinical score, indicating intermediate virulence. The Swine virus caused minimal to no weight loss, negligible respiratory signs, and no clinical deterioration. At the highest dose, differences between strains became more pronounced. The Turkey swine-like virus caused rapid and severe weight loss, with all mice succumbing by 5 dpi (Figure 2B). Respiratory distress was significantly more intense and persistent than at the lowest dose (Figure 2D). However, it seems that it did not fully account for lethality, as mice infected with the Turkey virus exhibited comparable respiratory impairment without mortality. Clinical scores in the Turkey swine-like virus group rapidly reached maximal values, confirming its highly virulent and lethal phenotype in mice (Figure 2F). In contrast, Turkey virus again induced moderate disease signs, including partial weight recovery and intermediate clinical scores, while the Swine virus remained largely nonvirulent, with only minor and transient effects. In addition, at the highest dose, only mice infected with the Turkey swine-like virus exhibited a marked and rapid drop in body temperature (Additional file 2), consistent with severe disease progression and lethality. Other groups maintained stable body temperatures throughout the course of infection.

Among the three strains tested, the Turkey swine-like virus displayed the highest virulence, characterized by rapid and severe weight loss, hypothermia, and 100% mortality. In contrast, the Turkey virus exhibited intermediate virulence, with transient morbidity and full recovery, consistent with a symptomatic but nonlethal infection. Finally, the Swine virus displayed minimal virulence in both the lowest and highest dose settings.

### Comparison of the replicative capacities of the different strains in mice

**Figure 3:**
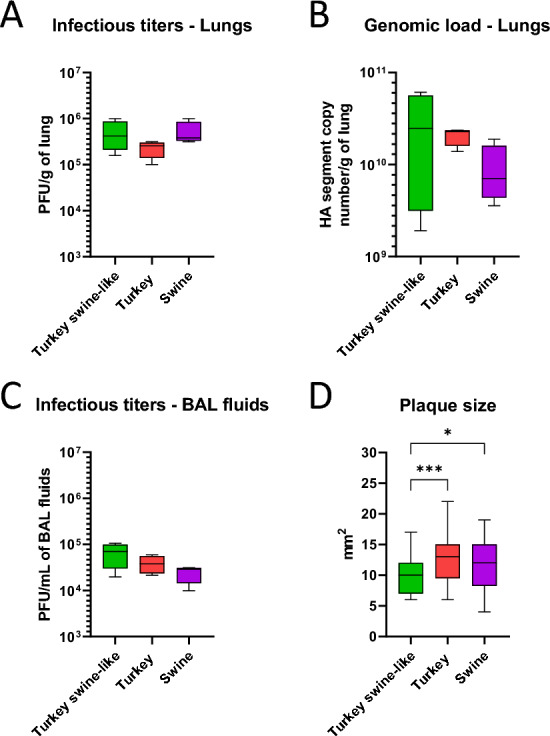
**Infectious titers, genomic load, and plaque size in infected BALB/c mice.** Mice were intranasally infected with the highest infectious dose (1.4 × 10^5^ PFU) of the three viral isolates at 3 dpi (*n* = 4). **A** Infectious viral titers in lung homogenates measured by plaque assay and expressed as PFU/g of lung tissue. **B** Viral genomic load in the lungs assessed by quantitative rRT-qPCR targeting the HA segment, expressed as copy number per gram of lung. **C** Infectious viral titers in BAL fluids measured by plaque assay and expressed as PFU/mL. **D** Plaque size (in mm^2^) measured in MDCK cells infected with the different viral isolates: *n* = 53 for Turkey swine-like virus plaques, *n* = 33 for Turkey virus plaques, and *n* = 28 for Swine virus plaques. Statistical significance was determined using one-way ANOVA. *p < 0.05, ***p < 0.001.

The rapid and early mortality observed in mice infected with the Turkey swine-like virus was particularly striking, especially in contrast with the Swine virus. To determine whether differences in viral replication could account for this phenomenon, we analyzed viral replication in the respiratory tract. We also analyzed viral replication in buccal swabs for the highest dose. As early as 1 day post-infection, high and early genomic loads were detectable, exceeding 10^4^ HA gene copies/mL in swabs for the Turkey swine-like virus and the Swine virus, and more than 10^3^ HA gene copies/mL in swabs for the Turkey virus (Additional file 3). Concerning the lung samples, we focused on the high-dose, death-inducing infection condition at 3 dpi, just prior to the onset of mortality in Turkey swine-like virus-infected mice. Infectious titers measured in lung homogenates showed that infectious viruses of all three viruses were produced at similar loads in the lungs (Figure 3A). Although the Turkey virus showed a slightly lower trend, differences were not statistically significant. Interestingly, quantification of viral genomic load in lung tissue on the basis of HA segment copy number (Figure 3B) revealed a significantly broader distribution for the Turkey swine-like virus, suggesting greater inter-individual variability in replication efficiency. Nonetheless, median genomic loads were comparable across strains. In BAL fluids (Figure 3C), infectious titers were slightly higher for the Turkey swine-like virus, but overall similar among the three strains, indicating efficient release of infectious particles into the lower airways regardless of strain. Finally, the plaque assay (Figure 3D) showed that the Turkey swine-like virus produced significantly smaller plaques in MDCK cells compared with both the Turkey and Swine strains, suggesting reduced in vitro cell-to-cell spread.

### Analysis of host inflammatory response in lungs of mice

**Figure 4:**
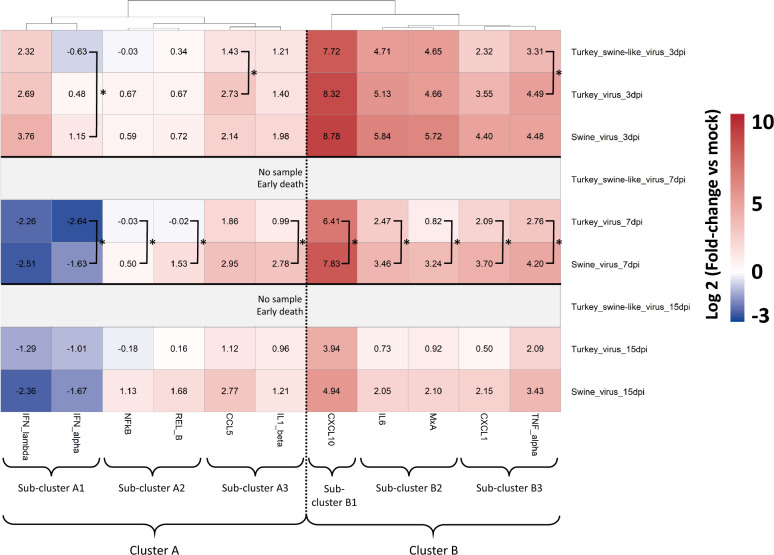
**Host immune response in lungs of infected BALB/c mice.** Mice were intranasally infected with the highest infectious dose (1.4 × 10^5^ PFU) of the three viral isolates and host immune response was measured at 3, 7, and 15 dpi (*n* = 4). 0 (white): gene expression level comparable to mock. Positive values (red): gene overexpression compared with mock. Negative values (blue): gene reduced expression compared with mock. Statistical significance was determined using the Mann–Whitney test. *p < 0.05. The two values in parentheses correspond to the minimum and maximum values.

To compare the host inflammatory response between the different viral strains used, we performed rRT-qPCR assays targeting the mRNA transcripts of 11 inflammation-related genes. This analysis focused on mice inoculated with the highest infectious dose. Heatmap clustering grouped genes according to their overall expression similarity across conditions (Figure 4). Genes with similar expression profiles clustered together in the dendrogram, forming visually coherent groups on the heatmap. Two main clusters were identified. The first cluster included genes showing moderate expression or reduced expression compared with the controls (cluster A, left part of the heatmap). Within this cluster, three sub-clusters were identified: the first included IFNλ and IFNα, with a slight overexpression at 3 dpi but reduced expression at 7 and 15 dpi in the Turkey virus and Swine virus groups; the second included NF-kB and RELB, with moderate expression levels close to controls; the third included CCL5 and IL1β, showing slight overexpression. The second main cluster included genes overexpressed relative to the controls (cluster B, right part of the heatmap). It also comprised three sub-clusters: the first contained CXCL10, which was strongly overexpressed at all time points post-infection; the second included IL6 and MxA, with overexpression mainly at 3 dpi; and the third included CXCL1 and TNFα, which showed slightly lower overexpression at 3 dpi but slightly higher at 7 dpi compared with the second sub-cluster.

At 3 dpi, the Turkey swine-like virus induced a lower expression of all genes compared with the Turkey virus (significantly for CCL5 and TNFα) and to the Swine virus (significantly for IFNα). Interestingly, at 7 dpi the Swine virus induced significantly higher expression of eight genes (NF-kB, RELB, IL1β, CXCL10, IL6, MxA, CXCL1, and TNFα) out of the nine overexpressed compared with the Turkey virus. The same trend was observed at 15 dpi for the nine overexpressed genes, although differences were not statistically significant (Figure 4). At 8 days post-infection, viral genomic loads in buccal swabs were still relatively high for the Turkey virus (more than 10^3^ HA gene copies/mL of swab), whereas no detection was observed for the Swine virus (Additional file 3). This suggests that the inflammatory response allowed control of viral replication for the Swine virus.

### Characterization of the inflammatory response using NF-κB luciferase reporter mice

**Figure 5:**
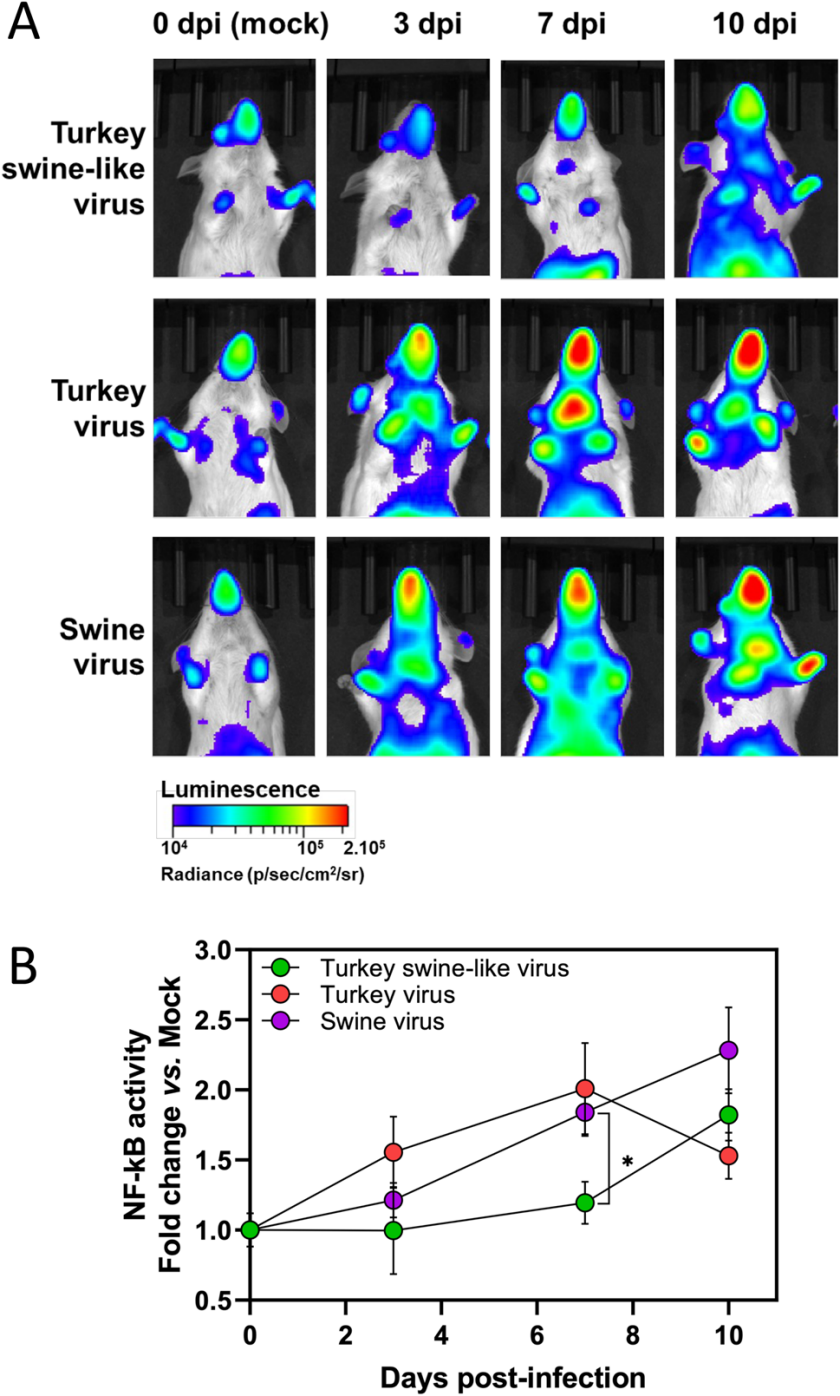
**In vivo maging of NF-κB activation in luciferase reporter mice following viral infection. **Transgenic mice expressing luciferase under the control of NF-κB-responsive elements were infected with 1.4 × 10^5^ PFU (highest infectious dose) of Turkey swine-like virus, Turkey virus, Swine virus, or mock-infected (*n* = 6). Bioluminescence was assessed by in vivoimaging on day 3, 7, and 10. **A** Representative images from each group showing the intensity and distribution of NF-κB-driven luciferase expression. The scale on the bottom indicates the average radiance: number of photons per second per square centimeter per steradian (p s^−1^ cm^−2^ sr^−1^). **B** Quantification of total luminescence in individual animals across the experimental groups: Turkey swine-like virus (green), Turkey virus (red), and Swine virus (purple). Data are presented as mean ± SEM. Statistical significance was determined using the Mann–Whitney test. *p < 0.05.

Analysis of the host response revealed a central role for inflammation mediated by the transcription factor NF-κB. The NF-κB-driven response was particularly distinctive and enabled discrimination between infected groups. Mice infected with the Turkey swine-like or Turkey viruses showed a reduced or delayed NF-κB response compared with mice infected with the Swine virus, in which infection induced a slight overexpression of the NF-κB gene. To further investigate this phenomenon, we quantified and compared the dynamics of NF-κB activation using luciferase reporter mice. Bioluminescence imaging at 3 and 7 dpi on mice infected with the highest infectious dose (1.4 × 10^5^ PFU) of each virus revealed marked differences in the intensity of NF-κB activation between groups (Figure 5A). Mice infected with the Turkey and Swine viruses exhibited strong and widespread signals, particularly in the upper respiratory tract consistent with mucosal immune activation. In contrast, mice infected with the Turkey swine-like virus showed minimal or no detectable inflammation at these early time points. However, by 10 dpi, luminescence signals in the Turkey swine-like group increased substantially, reaching levels comparable to those observed in the other two groups at 3 dpi. The quantitative assessment of the inflammatory response was provided by measuring the total photon flux emitted by each animal (Figure 5B). This dynamic representation confirmed the delayed induction of NF-κB activity in mice infected with the Turkey swine-like virus, as compared with those infected with the Turkey or Swine viruses. Notably, at 7 dpi, the differences in NF-κB activation between groups were statistically significant, highlighting a distinct temporal pattern of host responses in the Turkey swine-like group.

### Histopathological analyses of mouse lungs

**Figure 6:**
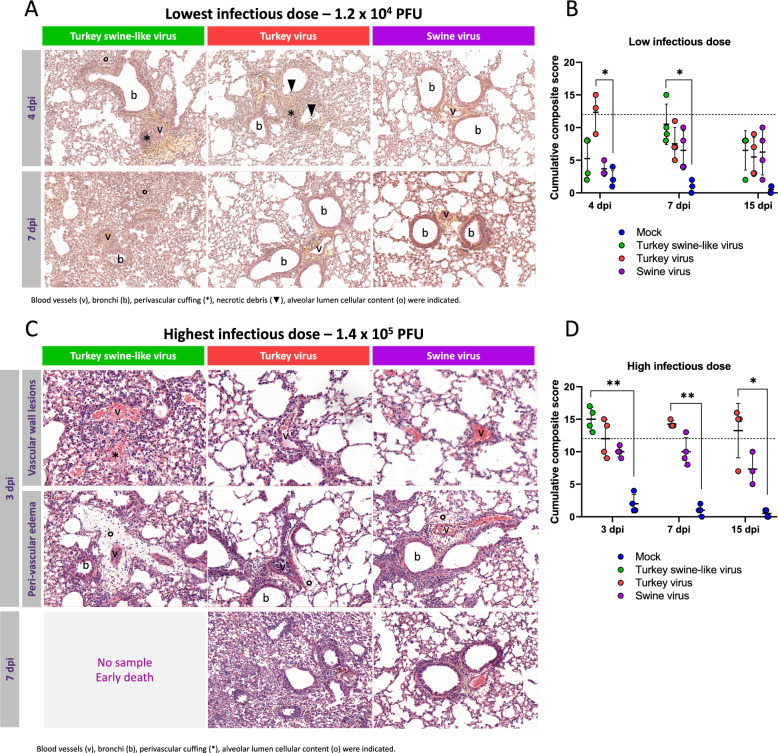
**Histopathology of lungs from infected BALB/c mice.** Mice were intranasally infected with three viral isolates. **A** Lung sections stained with HES at 4 and 7 dpi following the lowest infectious dose (1.2 × 10^4^ PFU). Blood vessels (v), bronchi (b), perivascular cuffing (*), necrotic debris (black arrowhead), and alveolar lumen cellular content (o) are indicated. **B** Cumulative composite score evolution over time for the lowest infectious dose (*n* = 4). Lung sections were scored on the basis of the following eight parameters: bronchial epithelium damage, bronchial content, vascular wall changes, perivascular edema, peribronchial/perivascular cuffing, interstitial pneumonia, alveolar content, and emphysema. A score > 12 (dashed line) indicated severe lesions. **C** Lung sections stained with HES at 4 and 7 dpi following the highest infectious dose (1.4 × 10^5^ PFU). Blood vessels (v), bronchi (b), severe hemorrhages (*), and perivascular edema (o) are indicated. **D** Cumulative composite score evolution over time for the highest infectious dose, on the basis of the same eight parameters (*n* = 4) for all conditions except Swine virus at 15 dpi where *n* = 3. A score > 12 (dashed line) indicated severe lesions. ND indicates that no tissues were available for the Turkey swine-like virus after 3 dpi owing to early death. Statistical significance was determined using Kruskall Wallis test with Dunn’s multiple comparisons test. *p < 0.05, **p < 0.01.

No macroscopic lesions were detected in any of the lung lobes. To compare lung lesions induced by the three viral strains, histopathological analyses were performed on lung samples collected from mice inoculated with the lowest infectious dose (1.2 × 10^4^ PFU) or the highest infectious dose (1.4 × 10^5^ PFU). All three viruses induced typical pneumonia lesions, with severity varying by strain, individual, and time post-infection (Figure 6). Elementary lesions were then scored and a composite total score was calculated to reflect the global intensity of broncho-interstitial pneumonia.

With the lowest dose, at 4 dpi, lesions were more pronounced with the Turkey virus, showing more extensive infiltrates than with the Turkey swine-like virus, in which only the perivascular parenchyma was affected (Figure 6A). At 7 dpi, however, lesion dynamics diverged: they markedly worsened with the Turkey swine-like virus, while their severity decreased with the Turkey virus. Nevertheless, at 7 dpi with the lowest dose, no significant difference was observed between these two viruses owing to individual variability. At both 4 and 7 dpi, lesions remained less severe following infection with the Swine virus. The peak in lesion scoring was reached as soon as 4 dpi for the Turkey virus and later, at 7 dpi, for the Turkey swine-like virus with two out of three and one out of four individuals showing a composite score > 12, respectively (Figure 6B). Lesions were similar over time for the Swine virus.

Following high-dose inoculation, at 3 dpi, the Turkey swine-like virus induced more severe vascular lesions than the Swine virus and the Turkey virus, with vascular wall damage and leakage, leading to marked hemorrhages and perivascular edema (Figure 6C). Regarding cumulative composite scores, lesions were more severe across all groups compared with low-dose inoculation (Figure 6D). Severe lesions (composite score > 12) were systematically observed with Turkey swine-like virus at 3 dpi and were frequent with Turkey virus at 3, 7, and 15 dpi (respectively *n* = 2/4, 4/4, and 3/4) but were only observed once with Swine virus at 7 dpi.

### Analysis of anti-NP immunostaining in mouse lungs

**Figure 7:**
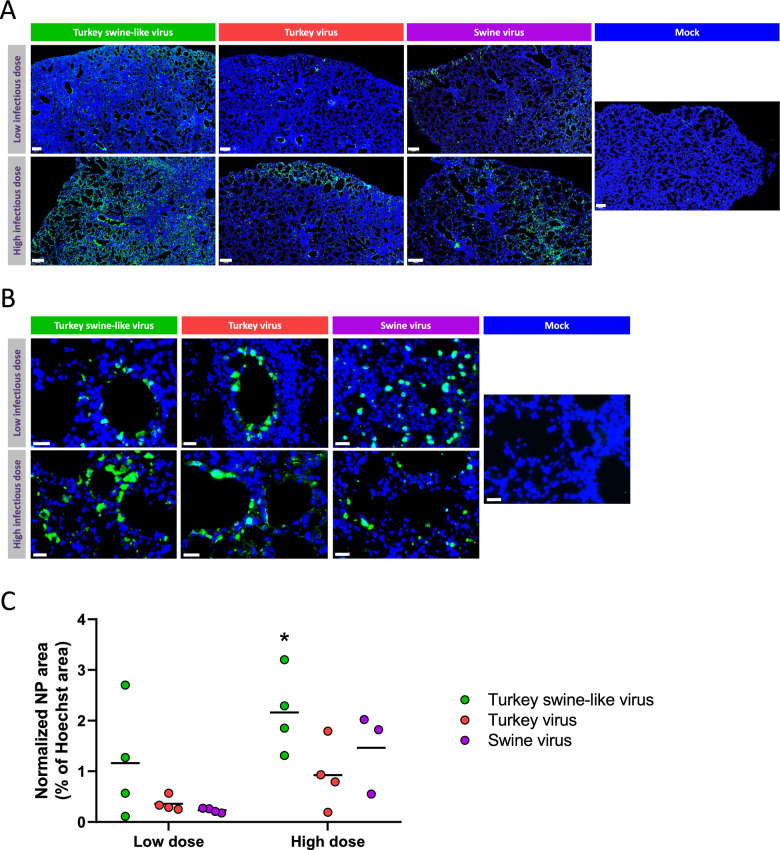
**Immunostaining of lungs from infected BALB/c mice. **Mice were intranasally infected with the highest infectious dose (1.4 × 10^5^ PFU) and the lowest infectious dose (1.2 × 10^4^ PFU) of the three viral isolates and immunostaining was performed at 4 and 3 dpi, respectively (*n* = 4). Blue signal: Hoechst staining, corresponding to cell nuclei. Green signal: NP protein, corresponding to IAV. **A** Overview of lung sections for the three viral conditions and the control group for the lowest dose (first row) and the highest dose (second row). Scale bar = 200 µm. **B** Close-up of preferential viral localization for the three viral conditions and the control group for the lowest dose (first row) and the highest dose (second row). Scale bar = 25 µm. **C** Normalized NP area expressed as a percentage of nuclear surface area, measured on whole lung sections for the three viral conditions for both doses (*n* = 4 for the lowest dose, *n* = 4 for the highest dose for all conditions except Swine virus where *n* = 3). Horizontal bars indicate the mean values. Statistical significance between doses was assessed using the Mann–Whitney test. Statistical significance between viruses and mock was assessed using the Kruskal–Wallis test followed by Dunn’s multiple comparisons test. *p < 0.05.

To compare both quantity and distribution of the virus within lung tissue, anti-NP immunostainings were performed on lung sections from mice infected with either the lowest or the highest infectious dose. For all three viral conditions and at both doses at 3 dpi, viral presence was observed in lung tissues compared with the mock condition (Figure 7A). As expected, the viral signal was stronger with the higher dose for all three viruses, with a marked increase for the Swine virus compared with the lowest dose (Figure 7A and C). Comparison of the area covered by the NP protein signal (green) normalized to the area occupied by cell nuclei (blue) showed that for both doses, a higher viral presence was detected in lungs of mice infected with the Turkey swine-like virus (Figure 7C).

Regarding the distribution of the virus within the lung tissue, the Turkey swine-like virus and the Turkey virus appeared more frequently in the bronchial epithelium compared with the Swine virus, which seemed to display a more alveolar tropism (Figure 7B). This difference was more apparent with the lowest infectious dose, while at the highest dose the viral signal was more frequently observed in the bronchial epithelium. At 7 dpi, the NP-associated signal markedly decreased and was very weak in all three inoculated groups (data not shown).

### Cytospins and MPO activity in BAL fluids

**Figure 8:**
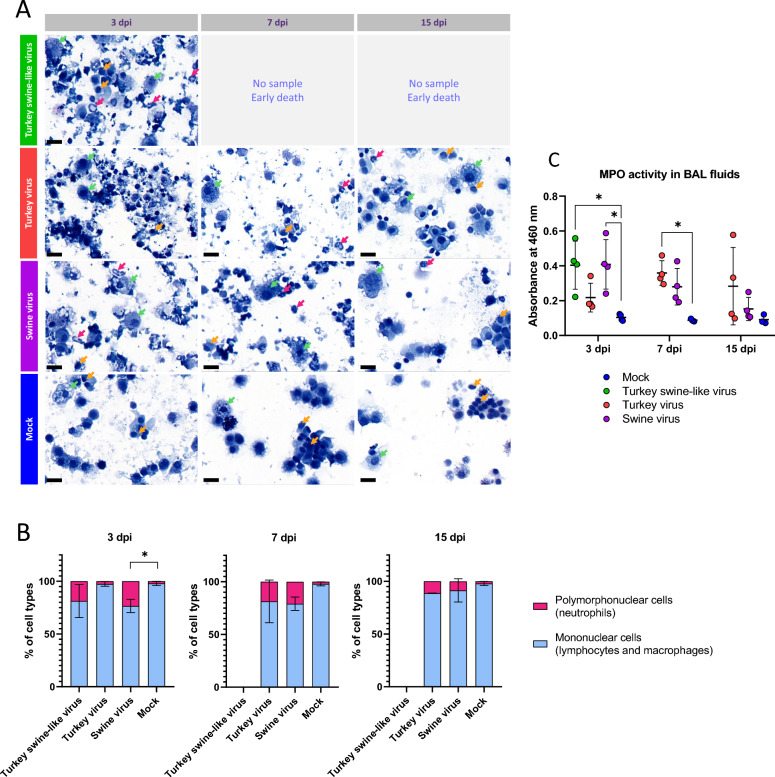
**Characteristics of BAL cells and supernatants from infected BALB/c mice.** Mice were intranasally infected with the highest infectious dose (1.4 × 10^5^ PFU) of the three viral isolates and immune cell counts in the BAL fluids were performed at 3, 7, and 15 dpi (*n* = 4). **A** Cytospins of immune cells stained with May–Grünwald Giemsa for the three viral conditions and the control group. Pink arrows: neutrophils. Orange arrows: lymphocytes. Green arrows: macrophages. Scale bar = 20 µm. **B** Percentage of polymorphonuclear cells (neutrophils; pink) and mononuclear cells (lymphocytes and macrophages; light blue) for the three viral conditions and the control group. **C** MPO activity in BAL fluids for the three viral conditions and the control group. Data are presented as mean ± SD. Statistical significance was assessed using the Kruskal–Wallis test followed by Dunn’s multiple comparisons test. *p < 0.05.

BAL fluids analysis in mice infected with the highest infectious dose (1.4 × 10^5^ PFU) was performed at 3, 7, and 15 dpi. BAL cells were centrifuged onto slides and stained with MGG. MPO activity was measured using the o-dianisidine substrate. At 3 dpi, a higher proportion of neutrophils (Figure 8A and B, pink arrows and pink bars) was observed in mice infected with the Turkey swine-like virus and the Swine virus compared with controls and to mice infected with the Turkey virus (Figure 8B). At 7 dpi, a delayed inflammatory response was observed in mice infected with the Turkey virus, with a higher proportion of neutrophils (Figure 8A and B, pink arrows and pink bars) compared with 3 dpi and to controls. At 15 dpi, this delayed response persisted in the Turkey virus group, with sustained higher proportions of neutrophils compared with controls. In mice infected with the Swine virus, at 7 dpi, the proportion of neutrophils remained similar to 3 dpi and to mice infected with the Turkey virus. At 15 dpi, the proportion of neutrophils decreased compared with 3 and 7 dpi and was also similar to mice infected with the Turkey virus (Figure 8B).

Regarding MPO activity in BAL fluids, a significantly higher absorbance was observed at 3 dpi in the Turkey swine-like virus and Swine virus groups compared with controls (Figure 8C), consistent with the increased neutrophil proportions. At 7 dpi, MPO activity was significantly higher in the Turkey virus group compared with controls, confirming the delayed inflammatory response observed (Figure 8B and C). At 15 dpi, MPO levels remained elevated in the Turkey virus group although without statistical significance, while they returned to baseline in the Swine virus group.

## Discussion

Following the multiple cross-species transmission events of a new IAV genotype A(H1_av_N2#)E from swine to turkeys, as well as a human case, it was of interest to study this virus both in turkeys and in a mammalian model. On the basis of phylogenetic analyses, three viral strains were selected for this study, representative of the circulating strains detected. The Turkey swine-like virus was the first strain to be identified in turkeys and was phylogenetically close to swine sequences. The Turkey virus was selected from a phylogenetic cluster composed exclusively of turkey sequences and appeared to result from viral selection and adaptation in this species [22]. The Swine virus had been isolated from pigs and was a reference strain previously studied in this host [23]. In pigs, infection with the swine H1_av_N2#E virus was associated with more severe clinical signs, earlier and higher viral shedding than previous major enzootic lineage swine A(H1_av_N1), and exhibited limited cross-neutralization with A(H1_av_N1), despite evidence of cross-reactive cell-mediated immunity. In our study, in turkeys inoculated with the Swine virus, clinical signs and viral shedding were negligible, whereas in mice, infection resulted in high viral replication in the lungs but only mild clinical manifestations with a robust immune response. Together, these results highlighted the contrasting host adaptation and immune dynamics of this virus, suggesting that pathogenicity and replication may differ substantially between swine, avian, and murine hosts.

The in vivo experiment conducted on 34-week-old breeding turkeys revealed few clinical signs across all three groups, with some birds displaying rales, respiratory distress, and lethargy. These signs appeared slightly earlier in the group infected with the Turkey swine-like virus (3 dpi), compared with the Swine virus (4 dpi) and the Turkey virus (7 dpi). Turkeys infected with the Turkey swine-like virus lost an average of 6.5% of their initial body weight by 10 dpi, those infected with the Turkey virus lost 4.7%, and no weight loss was observed in the group infected with the Swine virus. Despite these mild clinical signs, no viral genome was detected in the organs of any group. Viral shedding in oropharyngeal and cloacal swabs was nearly absent, with only a few sporadic detections and very low viral genomic loads. At 10 dpi, serological analyses showed that two out of four animals in the Turkey swine-like virus and Swine virus groups, and one out of four in the Turkey virus group, were seropositive for anti-NP antibodies. These results were somewhat unexpected compared with field observations, where, although the only clinical sign reported was a drop in egg production, clear viral shedding was observed from both trachea and cloaca. Two main hypotheses may explain these results. First, the ocular and nasal inoculation routes used may not have been optimal. Several studies have demonstrated that aerosol transmission, particularly via infected microdroplets, is a highly efficient route for IAV infection [31, 32]. Aerosol inoculation may have led to more efficient infection in turkeys. This method has already been used successfully in mouse and swine models [33, 34]. Alternatively, intratracheal inoculation could also be considered, as this route has proven effective in previous studies in turkeys, chickens, mice, and pigs [23, 35–37]. Second, potential co-infection with other pathogens in the field could explain the limited clinical signs observed in turkeys under experimental conditions. Sequencing analyses of the viral inocula confirmed the presence of pure IAV. We cannot exclude the concurrent circulation of other pathogens such as *Mycoplasma* spp., *Salmonella* spp. or *Pasteurella* spp. in breeding turkey flocks that could trigger clinical signs in affected farms. In livestock, bacterial co-infections have been shown to increase the pathogenicity of IAVs, especially low pathogenic avian influenza (LPAI) strains [38, 39]. The clinical signs and pathogenicity may be exacerbated through converging mechanisms such as epithelial damage and impaired mucociliary clearance facilitating bacterial adhesion, type-I-IFN-driven impairment of antibacterial innate immunity, and protease-mediated activation of HA that amplifies local replication [40–42]. These co-infections, in association with IAV, may have contributed in the field to the observed drop in egg production, which we could not reproduce in our experimental setting through inoculation of pure H1_av_N2#E viral strains.

On the contrary, in the mouse model, all three viruses caused substantial clinical signs and lung pathology, but their severity followed the hierarchy: Turkey swine-like virus > Turkey virus > Swine virus, regardless of the infectious dose. This difference was most striking at the highest dose (1.4 × 10^5^ PFU), where the Turkey swine-like virus infection led to rapid disease progression, requiring euthanasia of all mice between 5 and 6 dpi. As expected, the dose strongly influenced outcomes, with more severe lesions at the highest infectious dose for all viruses. At the lowest dose, the Turkey virus induced more lung lesions at 4 dpi, whereas at 7 dpi for the lowest dose and at 3 dpi for the highest dose, the Turkey swine-like virus consistently caused the most pronounced pathology. Interestingly, despite these marked differences in pathogenicity, no major differences in replication or viral shedding were detected between the three strains, all replicating efficiently in the lungs. Previous replication kinetics in mammalian cells and embryonated chicken eggs also indicated minimal differences in replication and infectious virus production among the three viruses [22]. Beyond these minimal differences, none of the three viruses caused mortality in embryonated eggs, further supporting their limited pathogenicity in this model. All these results argue against replication efficiency being the primary determinant of the distinct mortality and morbidity profiles observed in mice and this suggested that factors other than viral load contributed to the observed severity in pathogenicity. Immunohistochemistry indicated a potential difference in respiratory tropism, particularly at the lowest dose, with the Turkey swine-like virus and the Turkey virus preferentially localized to peribronchial regions, whereas the Swine virus appeared more frequently in alveolar areas.

Analysis of the host inflammatory response revealed two main expression profiles among the three viruses, with cluster A containing genes with low or moderate expression (IFNα/λ, NF-κB, RELB, CCL5, and IL1β) and cluster B containing genes with more pronounced overexpression (CXCL10, IL-6, MxA, CXCL1, and TNFα). In cluster A, type I and type III interferons (IFNα and IFNλ) displayed a transient overexpression at 3 dpi followed by reduced expression at 7 and 15 dpi, suggesting an early but short-lived activation of the classical antiviral pathway [43, 44]. In cluster B, CXCL10, an IFN-induced chemokine that promotes T and NK cell recruitment was strongly overexpressed at all time points, consistent with its key role in shaping adaptive immunity [45]. The other four genes in this cluster (IL-6, MxA, CXCL1, and TNFα) showed stronger overexpression at 3 dpi, reflecting a rapid antiviral response, with MxA being a major interferon-stimulated gene blocking IAV replication [46]. Comparison between viruses showed that the Turkey swine-like virus generally induced a weaker or delayed transcriptional activation that could partly explain the rapid mortality observed. Indeed, NF-κB activity in transgenic mice indicated a clear delay in the onset of the host inflammatory response following infection with the Turkey swine-like virus, highlighting potential differences relative to immune evasion mechanisms. A weaker innate response may have led to earlier and stronger clinical deterioration. Conversely, the Swine virus triggered the strongest overexpression for most genes, notably IFNα, NF-κB, RELB, IL1β, CXCL10, IL-6, MxA, CXCL1, and TNFα at 3 dpi and also significantly at 7 dpi. This profile suggested a more robust immune activation that, although associated with histopathological damage, may have helped to reduce clinical severity. The Turkey virus displayed an intermediate profile in terms of both clinical signs and inflammatory response. These transcriptional findings were consistent with immune cell dynamics in the BAL fluids, quantified using an AI-based application for automated cell identification. This approach saved substantial analysis time and enabled exhaustive counting of all cells in each cytospin spot. Neutrophil counts were higher at 3 dpi in Turkey swine-like virus- and Swine virus-infected mice compared with Turkey virus-infected mice, whereas the latter showed a greater neutrophil proportion at 7 dpi suggesting delayed recruitment. This pattern was mirrored by MPO activity levels in BAL fluids, which were elevated in Turkey swine-like virus and Swine virus infections at 3 dpi and in Turkey virus infections at 7 dpi. MPO, a peroxidase predominantly produced by neutrophils, has been shown to inactivate IAV [47, 48] and is known to be upregulated during infection [49]. In this study, the differences in mortality observed could be explained by three factors: a distinct tropism of the viruses following the swine-to-turkey species jump, whether recent or after adaptation; a delayed induction of the immune response; and a qualitatively different immune response. The results suggested that these three events were interconnected, and that their combined effect, particularly the timing and the intensity of the host response, ultimately drove the differences in virulence and pathogenicity observed.

Despite the differences in pathogenicity and induced immunity in mice, the three studied viruses shared a high level of genetic similarity. However, numerous factors beyond protein sequence variation may contribute to the observed differences. Synonymous mutations can alter RNA secondary structure or codon usage that can impact translation efficiency, molecular interactions, and viral fitness [50, 51]. Between the Swine virus and the Turkey swine-like virus, 16 synonymous mutations were identified across the whole genome. These were located in PB2 (positions 1839, 2166, and 2274), PB1 (positions 1683 and 2154), PA (positions 420 and 1128), NP (positions 51, 273, 507, and 543), NA (positions 321, 675, and 1098), M1 (position 258) and M2 (position 267). Mutations in noncoding regions, such as those in gene termini affecting packaging signals, can influence viral replication [52]. Post-translational modifications may also play a role, including *N*-glycosylation of HA which can alter antigenicity and virulence, or NP phosphorylation which regulates ribonucleoprotein (RNP) assembly and viral replication [53, 54]. Within a given host, IAVs circulate as quasispecies, which are populations of closely related but nonidentical genomes resulting from polymerase errors during replication [55]. Such minority variants could influence infection dynamics and pathogenic outcomes.

These marked differences in pathogenicity and induced immunity, particularly between the Swine virus and the Turkey swine-like virus, were somewhat unexpected, as only two nonsynonymous mutations were identified in the whole genome between these two viruses: K189R in the PB1 protein and E233K in the HA protein. It can therefore be hypothesized that one of these two mutations may be responsible for the substantial difference in the pathogenicity observed between the two strains. The E233K mutation is not located within the primer and probe binding regions and therefore did not affect rRT-qPCR efficiency.

The first nonsynonymous mutation, K189R in PB1, was located within the β-ribbon region (residues 177–212) of the N-terminal domain, a structural element involved in the PB1–PA interaction interface and containing two nuclear localization signals (NLS1 and NLS2) [56]. Residue 189 is part of NLS1 (residues 187–190), a basic region essential for binding to the cellular nuclear import factor Ran binding protein 5 (RanBP5) [57]. This interaction is necessary for the nuclear import of the PA–PB1 heterodimer and the subsequent assembly of the influenza polymerase complex [58]. While the K189R substitution retains a basic residue, it may alter the local charge distribution, the structural dynamics of the β-ribbon and potentially the affinity for RanBP5 that could in turn influence nuclear import efficiency and polymerase function. Position 233 on the hemagglutinin is located in the globular head of the HA1 subunit, within the 220-loop of the receptor binding site (RBS). This RBS domain is known to be a key determinant in HA binding to α2,3- or α2,6-linked SA and plays a role in infectivity [59, 60]. The substitution of an acidic amino acid (glutamate) with a basic amino acid (lysine) may alter the physicochemical properties of the protein. Such a change can modify the conformation and consequently the activity of the protein. For example, the E627K substitution in PB2 increases the catalytic rate of the polymerase at lower temperatures (around 34 °C) without altering its affinity, thereby enhancing replication in the human respiratory tract and promoting adaptation of avian viruses to mammals, especially humans [61]. In HA, the E47K mutation located in the HA2 subunit has been shown to stabilize the trimeric structure through a salt bridge (K24–E21), lowering the acidic threshold required for fusion and increasing both thermal and infectious stability [62]. Within the HA RBS, a mutation from an acidic to a basic amino acid can alter the electrostatic interaction network near the binding site and modify the local flexibility of the 220-loop, thereby affecting the opening and closing dynamics of the site. This may result in stronger or weaker interactions with SA and ultimately in changes in viral tropism [59, 63, 64]. In our study, three-dimensional (3D) structural predictions of the HA from the Swine virus and the Turkey swine-like virus show different steric hindrance (data not shown). This suggests a modification of the interaction between the RBS and cellular sialic acids. It has previously been shown that positions 233 and 236, corresponding to positions 222 and 225 in H1 numbering, are critical for cellular attachment of the H1N1 virus of the Spanish flu [65]. Our main hypothesis is that the HA E233K mutation between the Swine virus and the Turkey swine-like virus is responsible for the observed differences, playing a role in sialic acid binding and/or modulation of the inflammatory response. Indeed, since viral replication itself did not appear to be the discriminating factor between the three viruses, whereas the immune response did, the role of the RBS in shaping immunity warrants consideration. The HA, through its RBS, can bind to dendritic cells via C-type lectin receptors (CLRs) even in the absence of SA [66]. These CLRs can trigger intracellular signaling pathways that modulate cytokine production, including type I interferons (IFN-I) [67]. A structural change in the RBS could therefore alter HA affinity for CLRs, thereby influencing their role in the immune response. In addition, attachment of IAVs to the cell surface through interactions between the RBS and SA activates the epidermal growth factor receptor (EGFR), which modulates antiviral signaling pathways such as IFN production [68]. The RBS therefore acts not merely as an anchor but as an early modulator of the immune response upon host-cell binding. It has also been shown that HA alone can activate the transcription factor NF-κB [69]. Although the precise structural mechanism remains unclear, accumulation of HA proteins in the endoplasmic reticulum appears to drive NF-κB activation [70]. Furthermore, HA, particularly through its HA1 subunit, can induce phosphorylation, ubiquitination, and degradation of the IFNAR1 receptor, thereby reducing cellular sensitivity to type I IFNs [71]. This illustrates that HA also acts as an active modulator of the innate immune response by downregulating IFN signaling. While the RBS may not be directly involved in each of these mechanisms, structural modifications in HA, especially in the HA1 subunit that contains the RBS, may influence its intracellular interactions and activity, with downstream consequences for the regulation of inflammatory pathways. Taken together, these considerations support the hypothesis that the mutation observed at position 233 in HA is not only involved in receptor binding to SA but may also contribute to modulation of the inflammatory response and immune evasion.

The protein differences with the Turkey virus, which showed an intermediate profile between the Swine virus and the Turkey swine-like virus in terms of pathogenicity and induced immunity, were somewhat more substantial. There were 34 and 35 amino acid differences, respectively, out of the total 4789 amino acids of the complete proteome. Most differences were located in the HA protein, with eight amino acid substitutions, followed by NA with five differences, and PB1 with five and four substitutions compared with the Swine virus and the Turkey swine-like virus, respectively [22]. We can therefore hypothesize that the observed phenotypic differences may originate from one or several mutations in these proteins. At position 189 of PB1, the Turkey virus shared the same amino acid as the Turkey swine-like virus, an arginine (R). Interestingly, two mutations emerged in the Turkey virus in the HA protein after a single multiplication on MDCK cells: K233M and E236G. Although this avian-origin virus initially carried the same amino acid as the Turkey swine-like virus at position 233, the K233M substitution may have shifted it toward a more mammalian-like signature. This would be consistent with the intermediate clinical profile observed between a mammalian-origin virus (Swine virus) and an avian-origin virus (Turkey swine-like virus).

To further identify molecular markers of pathogenicity in these H1_av_N2#E viruses in mice, it would be interesting to examine the functional impact of the PB1 K189R and HA E233K substitutions and more particularly the mutation in HA in position 233. Generation of reverse genetics-derived mutant viruses carrying each mutation individually would allow identification of a potential virulence marker, while ensuring a single and homogeneous viral population to rule out the quasispecies effect. Finally, further studies could investigate factors beyond protein sequence differences, such as RNA secondary structures or post-translational modifications.

In conclusion, this study aimed at characterizing in turkeys and mice three H1_av_N2#E viruses: one isolated from swine, one turkey isolate originating from a recent swine-to-turkey spillover event, and one turkey isolate appearing adapted to this species. In breeding turkeys, infection resulted in minimal clinical signs and almost no detectable viral shedding. The two main hypotheses to explain this discrepancy with field observations are the use of a suboptimal inoculation route and the presence of bacterial or viral co-infections in the field, which could have exacerbated clinical signs. In mice, by contrast, all three viruses replicated, caused clinical disease and induced a differential immune response. The Turkey swine-like virus was the most virulent and induced the weakest immune activation, whereas the Swine virus showed the opposite pattern. The Turkey virus displayed an intermediate phenotype. Further investigation of the PB1 K189R and HA E233K substitutions may help determine whether one of these mutations is responsible for the observed differences in pathogenicity.

## Supplementary Information

**Additional file 1. Mutations among the three studied strains. First column: viral proteins with differences.** Second columns: two differences between the Swine virus and the Turkey swine like virus. Third column: 35 differences between the Swine virus and the Turkey virus and 34 differences between the Turkey swine-like virus and the Turkey virus. The two mutations in bold correspond to changes that emerged in the Turkey virus after propagation in MDCK cells.

**Additional file 2. Body temperature dynamics in infected BALB/c mice. **Mice (*n*=12 per group) were intranasally infected with either the lowest (1.2 × 10⁴ PFU, left panel) or the highest (1.4 × 10⁵ PFU, right panel) infectious dose of various IAV isolates: Turkey swine-like virus (green), Turkey virus (red), Swine virus (purple), or mock-infected (blue). Body temperature was monitored daily up to 14 days post-infection. From 0 to 3 dpi, *n*=12; from 4 to 7 dpi, *n*=8 and from 8 to 14 dpi, *n*=4. † indicates death of mice in the Turkey swine-like virus condition. Data are presented as mean ± SEM.

**Additional file 3. Viral genomic loads in the buccal swabs for the highest infectious dose in infected BALB/c mice. **Mice (n=12 per group) were intranasally infected with the highest (1.4 × 10⁵ PFU) infectious dose of various IAV isolates: Turkey swine-like virus (green), Turkey virus (red), Swine virus (purple), or mock-infected (blue). Viral genomic load in the buccal swabs were assessed at 1, 3, 6, 8, 10 and 14 dpi by quantitative rRT-qPCR targeting the HA segment, expressed as copy number per mL of buccal swab. From 0 to 3 dpi, *n*=12; from 4 to 7 dpi, *n*=8 and from 8 to 14 dpi, *n*=4. † indicates death of mice in the Turkey swine-like virus condition. Data are presented as mean ± SEM.

## Data Availability

The datasets used and analyzed during the current study are available from the corresponding authors on reasonable request.
