# Supplementary material for: Pathogenicity and immune response of turkey A(H1N2) influenza virus of swine-origin on turkeys and mice
Source: Vet Res. 2026 Jun 3;57:100. doi: 10.1186/s13567-026-01728-8 (PMC13235187; doi:10.1186/s13567-026-01728-8)
Supplement: Supplementary file 1 — Additional file 1. Mutations among the three studied strains. First column: viral proteins with differences. Second columns: two differences between the Swine virus and the Turkey swine like virus. Third column: 35 differences between the Swine virus and the Turkey virus and 34 differences between the Turkey swine-like virus and the Turkey virus. The two mutations in bold correspond to changes that emerged in the Turkey virus after propagation in MDCK cells. [file 13567_2026_1728_MOESM1_ESM.pdf]

Additional Table 1. Mutations among the three studied strains

Viral protein	Swine virus vs Turkey swine-like virus	Swine virus or Turkey swine-like virus vs Turkey virus
PB2	/	3 mutations:<br>I82S / R293Q / K508R
PB1	1 mutation:<br>K189R	5 mutations:<br>F94L / K or R189R / N375S / K388R / D752E
PB1-F2	/	2 mutations:<br>R13Q / S65Y
PA	/	1 mutation:<br>A448S
PA-X	/	1 mutation:<br>S237L
HA	1 mutation:<br>E233K	8 mutations:<br>F6V / N182I / E or **K233M** / **E236G** / E272D / S275Y / H287Y / I312F
NP	/	3 mutations:<br>T350A / A423V / V456M
NA	/	5 mutations:<br>V50I / S127G / E221K / T401N / S416N
NS1	/	4 mutations:<br>V65L / I137V / N176I / K217N
NS2	/	3 mutations:<br>M19L / S57F / N60T

First column: viral proteins with differences. Second columns: two differences between the Swine virus and the Turkey swine-like virus. Third column: 35 differences between the Swine virus and the Turkey virus and 34 differences between the Turkey swine-like virus and the Turkey virus. The two mutations in bold correspond to changes that emerged in the Turkey virus after propagation in MDCK cells.
