# Supplementary material for: Pathogenicity and immune response of turkey A(H1N2) influenza virus of swine-origin on turkeys and mice
Source: Vet Res. 2026 Jun 3;57:100. doi: 10.1186/s13567-026-01728-8 (PMC13235187; doi:10.1186/s13567-026-01728-8)
Supplement: Supplementary file 2 — Additional file 2. Body temperature dynamics in infected BALB/c mice. Mice (n=12 per group) were intranasally infected with either the lowest (1.2 × 10⁴ PFU, left panel) or the highest (1.4 × 10⁵ PFU, right panel) infectious dose of various IAV isolates: Turkey swine-like virus (green), Turkey virus (red), Swine virus (purple), or mock-infected (blue). Body temperature was monitored daily up to 14 days post-infection. From 0 to 3 dpi, n=12; from 4 to 7 dpi, n=8 and from 8 to 14 dpi, n=4. † indicates death of mice in the Turkey swine-like virus condition. Data are presented as mean ± SEM. [file 13567_2026_1728_MOESM2_ESM.pdf]

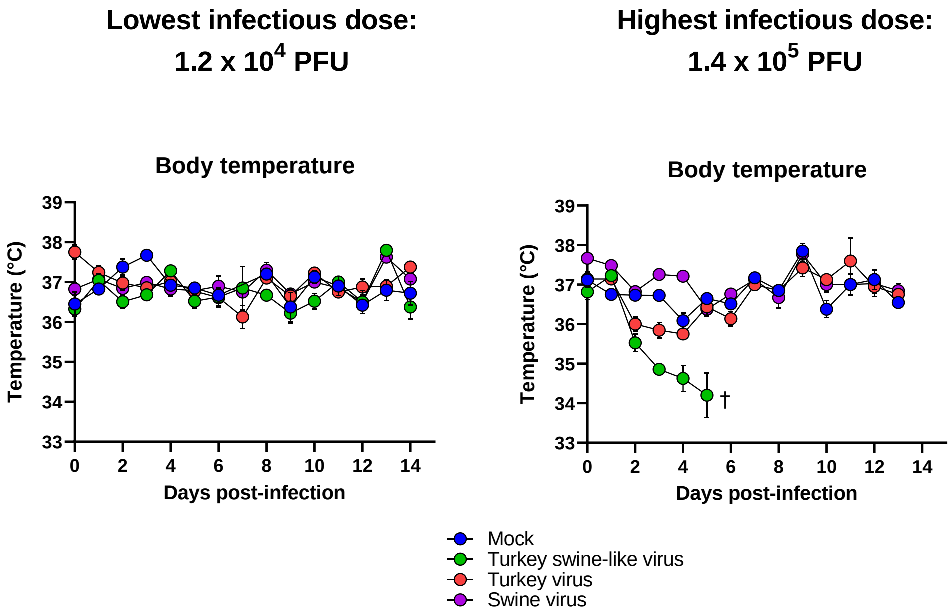

Lowest infectious dose:
1.2 x 10^4 PFU
Highest infectious dose:
1.4 x 10^5 PFU
Body temperature
Body temperature
Temperature (°C)
Temperature (°C)
39
38
37
36
35
34
33
0
2
4
6
8
10
12
14
Days post-infection
Days post-infection
†
Mock
Turkey swine-like virus
Turkey virus
Swine virus
