# Supplementary figures and images for: Pathogenicity and immune response of turkey A(H1N2) influenza virus of swine-origin on turkeys and mice
Source: Vet Res. 2026 Jun 3;57:100. doi: 10.1186/s13567-026-01728-8 (PMC13235187; doi:10.1186/s13567-026-01728-8)

## Viral genomic load in the buccal swabs

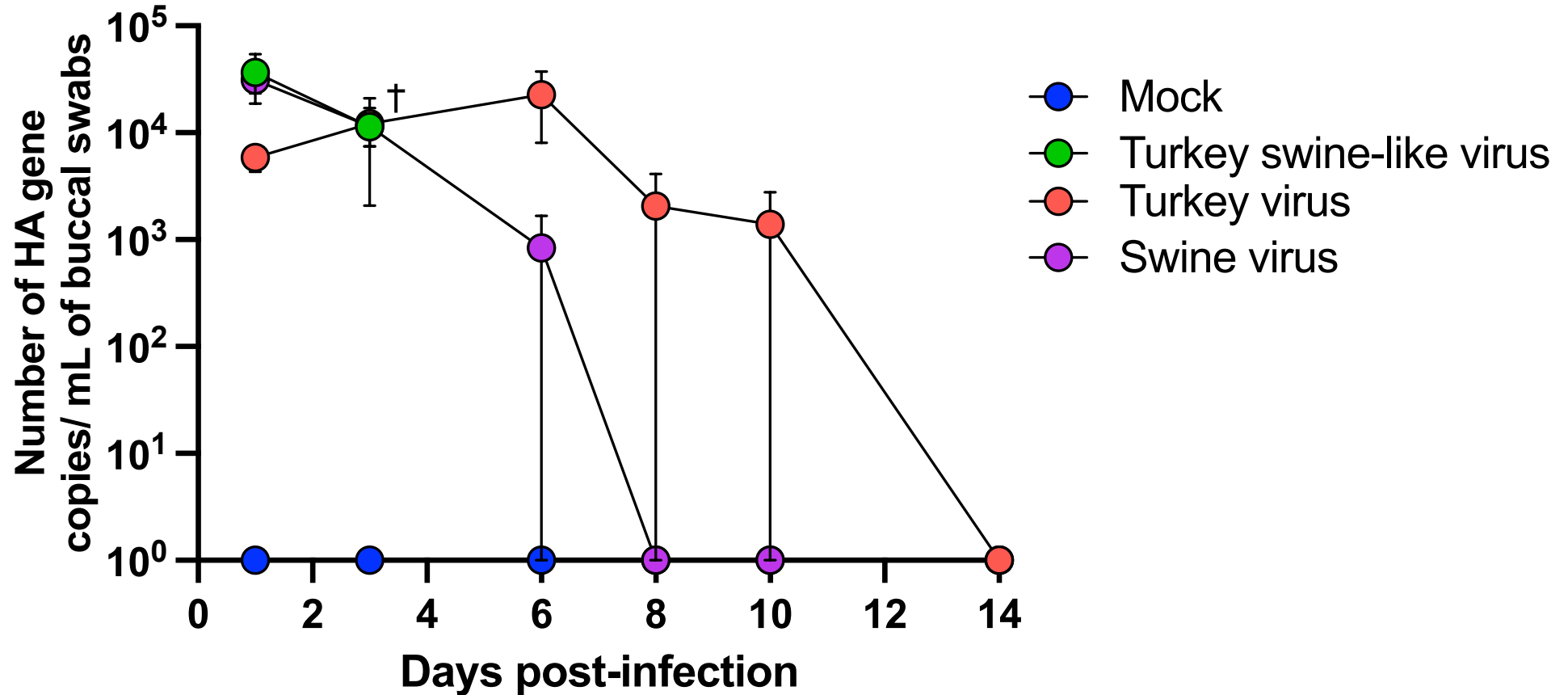

Supplement: Supplementary file 3 — Additional file 3. Viral genomic loads in the buccal swabs for the highest infectious dose in infected BALB/c mice. Mice (n=12 per group) were intranasally infected with the highest (1.4 × 10⁵ PFU) infectious dose of various IAV isolates: Turkey swine-like virus (green), Turkey virus (red), Swine virus (purple), or mock-infected (blue). Viral genomic load in the buccal swabs were assessed at 1, 3, 6, 8, 10 and 14 dpi by quantitative rRT-qPCR targeting the HA segment, expressed as copy number per mL of buccal swab. From 0 to 3 dpi, n=12; from 4 to 7 dpi, n=8 and from 8 to 14 dpi, n=4. † indicates death of mice in the Turkey swine-like virus condition. Data are presented as mean ± SEM. [file 13567_2026_1728_MOESM3_ESM.pdf]
